# Ischemic heart disease burden attributable to inadequate omega-3 fatty acid intake in Chinese adults, 1990–2021: an Age-Period-Cohort analysis of the Global Burden of Disease study

**DOI:** 10.3389/fnut.2025.1590278

**Published:** 2025-06-26

**Authors:** Lin Luo

**Affiliations:** ^1^School of Physical Education, Guizhou Normal University, Guiyang, China; ^2^Key Laboratory of Brain Function and Brain Disease Prevention and Treatment of Guizhou Province, Guiyang, China

**Keywords:** omega-3 fatty acids, ischemic heart disease, disease burden, Disability-Adjusted Life Years (DALYs), mortality

## Abstract

**Background:**

Insufficient omega-3 fatty acid intake is a significant modifiable risk factor for ischemic heart disease (IHD), yet its long-term impact on the disease burden in the Chinese population remains inadequately understood. This study, based on data from the Global Burden of Disease (GBD) study, is the first comprehensive analysis of the Age–Period–Cohort (APC) dynamics of IHD burden attributable to insufficient omega-3 intake in Chinese adults from 1990 to 2021, revealing age- and sex-related heterogeneity and social determinants.

**Methods:**

Data from the GBD 2019 study on Chinese adults aged 25–94 years, stratified by sex, were integrated. The age-period-cohort (APC) model was employed to analyze the spatiotemporal dynamics of death and disability-adjusted life years (DALYs), with net drift and local drift metrics used to quantify long-term trends.

**Results:**

In 2021, the death rate from IHD attributable to insufficient omega-3 intake demonstrated an exponential age gradient, with death increasing from 5.568 per 100,000 in the 25–29 age group to 3,806.93 per 100,000 in the 90–94 age group (a 684-fold increase). Male death (708.01 per 100,000) was significantly higher than female death (537.28 per 100,000), with sex differences following a nonlinear age pattern: male death was 54–78% higher than female death in younger and middle-aged adults, while female risk exceeded male risk in the 70–84 and 90–94 age groups. The DALY rate exhibited a critical turning point between the ages of 45 and 50 (30 → 475 per 100,000), with greater health risk heterogeneity observed among men (standard deviation 145.65 vs. 96.24 in women). From 1990 to 2021, the absolute number of deaths increased by 17.47%, while age-standardized death decreased by 19.72% (from 7.28 to 5.84 per 100,000), and the DALY rate declined by 41.40% (from 221.39 to 129.75 per 100,000). The APC model revealed: (1) Age effect: Death in the 90–94 age group reached 3,806.93 per 100,000, emphasizing the risks associated with extreme aging; (2) Period effect: A sharp 10% decrease in DALYs in the middle-aged group during 2005–2006, coinciding with strengthened nutrition policies; (3) Cohort effect: Death in the 1895–1994 birth cohort declined by 99.3%, reflecting progress in public health. Net drift analysis showed that the annual decline in DALY rate (−1.53%) was significantly greater than the death decline (−0.32%), with the most notable improvement in the middle-aged group (local drift of −3.87% per year).

**Conclusion:**

This study is the first to reveal the dynamic evolution of IHD burden attributable to insufficient omega-3 intake in China. Although both death rates and DALY rates have decreased, population aging continues to drive an increase in the absolute disease burden. By innovatively integrating Age–Period–Cohort modeling with nutritional epidemiology, this study provides multidimensional evidence to inform age- and sex-specific intervention strategies and to guide the refinement of dietary guidelines.

## 1 Introduction

Omega-3 fatty acids are essential polyunsaturated fatty acids that play a crucial role in maintaining cardiovascular health (1, 2). Epidemiological studies consistently indicate that insufficient omega-3 fatty acid intake may be a key nutritional risk factor for the onset and progression of ischemic heart disease (IHD) (3). Recent studies have shown that omega-3 fatty acid intake is generally low among Chinese adults (4), and despite an increase in vegetable oil consumption from 1997 to 2011, omega-3 fatty acid intake from marine sources remains insufficient (5).

IHD is a leading cause of death and disease burden worldwide (6). As the most populous country, China’s IHD burden warrants particular attention (7, 8). Large-scale clinical trials have confirmed that omega-3 fatty acid supplementation has significant cardiovascular protective effects (9). Studies have shown that omega-3 supplementation can significantly reduce the risk of major cardiovascular events (10, 11). Omega-3 fatty acids are known to have multiple protective effects, including suppressing inflammation, improving lipid profiles, reducing the risk of thrombosis, and regulating endothelial function (12–14).

China is undergoing a rapid nutritional transition, with changes in traditional dietary patterns leading to significant shifts in omega-3 fatty acid intake (5, 15). A study on marine omega-3 fatty acid intake indicated that the overall intake level among Chinese adults is low, which may be closely related to the risk of cardiovascular diseases (16). Between 1990 and 2021, China has experienced significant changes in socioeconomics, dietary structure, and lifestyle (17), which may have had a significant impact on omega-3 fatty acid intake and the IHD disease burden (18).

Spatiotemporal analysis methods provide a powerful framework for examining the dynamic changes in disease burden (19). The age-period-cohort (APC) model systematically decomposes the complex interactions between age, period, and cohort effects on disease burden (20), helping to deepen the understanding of the long-term evolution of omega-3 fatty acid intake and its impact on IHD.

Based on the Global Burden of Disease (GBD) study data, this research aims to comprehensively analyze the spatiotemporal evolution of the IHD burden attributable to insufficient omega-3 intake in Chinese adults from 1990 to 2021. The specific objectives of the study include: assessing the temporal trends in omega-3 fatty acid intake among Chinese adults from 1990 to 2021; quantifying the contribution of insufficient omega-3 intake to the IHD disease burden; and using the APC model to analyze the differential impact of omega-3 deficiency on IHD burden across different age groups, periods, and cohorts.

The innovation and significance of this study lie in: (1) the first systematic evaluation of the long-term impact of insufficient omega-3 intake on IHD among Chinese adults based on GBD data; (2) the use of Age-Period-Cohort analysis methods to reveal the complex relationship between omega-3 intake and IHD burden; and (3) providing scientific evidence to support the formulation of targeted nutritional intervention strategies and cardiovascular disease prevention policies.

## 2 Data and methods

### 2.1 Data sources

This study utilized data on ischemic heart disease (IHD) attributed to insufficient omega-3 fatty acid intake from the Global Burden of Disease Study 2021 (GBD 2021). The study sample includes the Chinese population aged 25–94 years, with a time span from 1990 to 2021. The GBD 2021 study employed a standardized framework to systematically analyze 369 diseases, injuries, and their sequelae across 204 countries and regions, while also evaluating the disease burden of 87 risk factors. For data collection in China, GBD 2021 integrated multiple sources, including the National Cause of Death Surveillance System, the Chinese Maternal and Child Health Surveillance Network, the Chinese Center for Disease Control and Prevention (CDC) cause of death reporting system, the National Nutrition and Health Survey data, and the National Chronic Disease Surveillance System. This study primarily focused on two disease burden indicators: Disability-Adjusted Life Years (DALYs) and mortality. Relevant data can be accessed through the official platform of the Institute for Health Metrics and Evaluation (IHME).^1^ Data extraction and analysis followed the standard methodology of GBD research to ensure the scientific rigor and reliability of the study.

### 2.2 Variables

#### 2.2.1 Omega-3 fatty acid intake levels

The Global Burden of Disease (GBD) study adopts a standardized nutritional assessment framework to systematically evaluate dietary risk factors, incorporating data collection tools such as 24-h dietary recalls and standardized data processing protocols. For inadequate intake of omega-3 fatty acids—including alpha-linolenic acid (ALA), eicosapentaenoic acid (EPA), and docosahexaenoic acid (DHA)—the GBD study synthesizes diverse data sources, including national nutrition and health surveys, dietary intake assessments, and biomarker analyses. Using the comparative risk assessment (CRA) framework, it quantifies the relationship between omega-3 intake and disease outcomes and determines the theoretical minimum risk exposure level (TMREL). A Bayesian hierarchical model is employed to address data heterogeneity, with targeted adjustments for age standardization, sex standardization, and seasonal variation. By integrating dietary data, biomarker levels, and recommended intake thresholds, the GBD ensures global comparability of omega-3 intake estimates, thereby strengthening the data foundation for dietary risk assessment.

#### 2.2.2 Ischemic heart disease burden

The disease burden of ischemic heart disease is measured using two indicators: Disability-Adjusted Life Years (DALYs) and mortality. DALYs are a comprehensive metric for disease burden, representing the years of healthy life lost due to premature death and disability. It is calculated as the sum of Years of Life Lost (YLLs) due to premature death and Years Lived with Disability (YLDs) due to disease. To account for societal preferences regarding the value of life at different ages, the GBD study incorporates a 3% annual time discount rate and an age-weighting function into the calculation of DALYs, such that future health losses are progressively discounted over time. Mortality refers to the total number of deaths attributed to ischemic heart disease during a specified period. This is estimated and adjusted using a standardized statistical model by integrating multiple data sources, including death registration systems, demographic data, and epidemiological surveys. This comprehensive approach ensures the accuracy and completeness of disease burden assessments.

### 2.3 Data analysis

#### 2.3.1 Time trend analysis

Statistical analysis was conducted using Python (version 3.9). A segmented regression model was employed to evaluate the time trend of Disability-Adjusted Life Years (DALYs) and mortality attributable to insufficient omega-3 fatty acid intake in ischemic heart disease (IHD) in China from 1990 to 2021. The Annual Percent Change (APC) was also calculated. The significance level for statistical tests was set at *α* = 0.05.

#### 2.3.2 Age-Period-Cohort model analysis

In the data processing of the Age-Period-Cohort (APC) model, the research team developed a rigorous data preprocessing workflow using Python. Key steps in the data processing included: extracting age information from the raw data with precision using Pandas’ regular expression and string manipulation functions; calculating the midpoint age for each record; and grouping the data into 14 age categories (25–29 years, 30–34 years, and so on up to 90–94 years) based on the rule: Age group = [Age midpoint ÷ 5] × 5. Using Pandas’ date processing functions, the years were divided into fixed 5-year intervals, and the start year for each period was calculated as: Year start = Year − [(Year − 1990) mod 5], resulting in 7 distinct period groups. In the final stage of data processing, the birth cohort was calculated using the formula: Birth cohort = Period midpoint − Age midpoint for each record, with re-grouping by 5-year intervals, yielding 20 cohort groups.

For the APC model analysis, the research team chose to use the Generalized Linear Model (GLM) and Poisson regression model from the statsmodels library. A complex design matrix was constructed to decompose the age, period, and cohort effects. Specifically, age, period, and cohort were treated as categorical variables in the model, using dummy variable encoding, and multicollinearity issues were controlled through constraint conditions. During model fitting, a Poisson distribution with a log link function was used to accommodate the count-type nature of the dependent variable. Model diagnostics were performed through residual analysis, information criteria (AIC/BIC), and over-dispersion tests to ensure the robustness of the model estimates. This meticulous data processing and modeling strategy allowed for a systematic decomposition of the age, period, and cohort effects of insufficient omega-3 fatty acid intake on the disease burden of ischemic heart disease, providing a scientific analytical framework for understanding the spatiotemporal evolution of the disease burden.

## 3 Results and analysis

### 3.1 Disease burden of ischemic heart disease attributable to insufficient omega-3 fatty acid intake in China in 2021

#### 3.1.1 Death rate due to ischemic heart disease attributable to insufficient omega-3 fatty acid intake in 2021 by gender and age group

This study systematically analyzed the age and gender distribution characteristics of Deaths due to ischemic heart disease (IHD) attributable to insufficient omega-3 fatty acid intake in China, revealing the complex dynamics of the disease burden. The results showed a significant age-dependent gradient in Deaths, with rates rising dramatically from 5.568 per 100,000 in the 25–29 age group to 3,806.93 per 100,000 in the 90–94 age group, an increase of nearly 684 times, reflecting a typical exponential growth pattern. Gender analysis revealed more detailed epidemiological features: the average Death rate in males (708.01 per 100,000) was significantly higher than that in females (537.28 per 100,000), but the gender differences across age groups were not a simple linear pattern.

Specifically, during the young and middle-aged period (30–50 years), male Death rates remained consistently significantly higher than those of females, with differences ranging from 54 to 78%. As individuals entered the middle-aged and elderly phase (50–69 years), the gender gap in Death rates gradually narrowed. In the 70–84 age group, a slightly higher Death rate in females was observed, while in the 90–94 age group, male Deaths again significantly exceeded those of females. The study identified several key inflection points: the accelerated growth period of Deaths between 50–69 years, the critical threshold of 50 per 100,000 reached in the 75–79 age group, and the crossing of the 200 per 100,000 threshold in the 85–89 age group.

**Figure 1:**
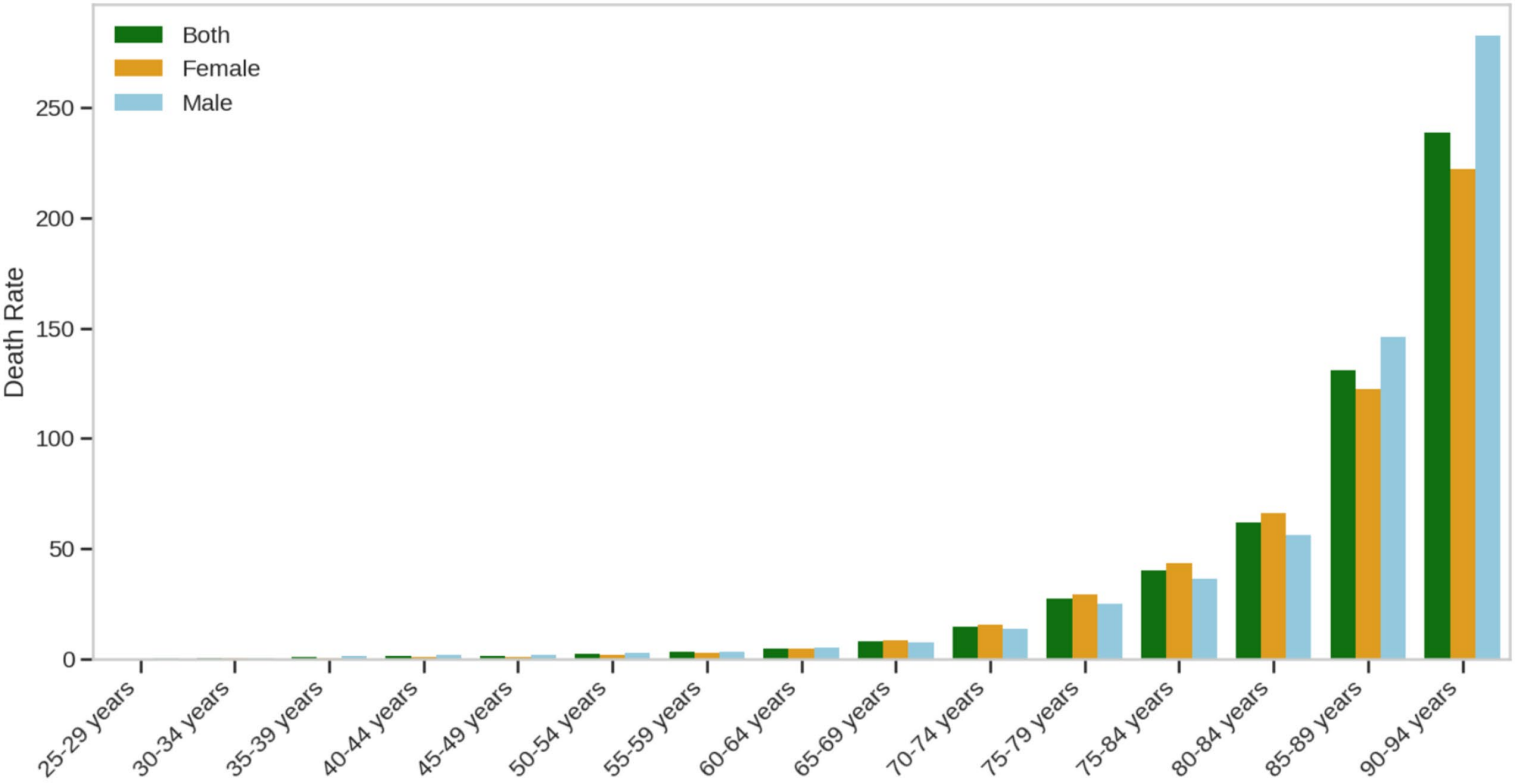
Death rate due to ischemic heart disease attributable to insufficient omega-3 fatty acid intake in 2021 by gender and age group.

The distribution characteristics of the data further revealed the complexity of Deaths. All three gender groups showed a severely right-skewed distribution, with extreme values in the older age groups significantly elevating the average levels. The male group exhibited the largest standard deviation (145.65 per 100,000), reflecting a high degree of individual variability in Death rates, a finding that is crucial for personalized health interventions (Figure 1).

#### 3.1.2 DALY rates due to ischemic heart disease attributable to insufficient omega-3 fatty acid intake in 2021 by gender and age group

**Figure 2:**
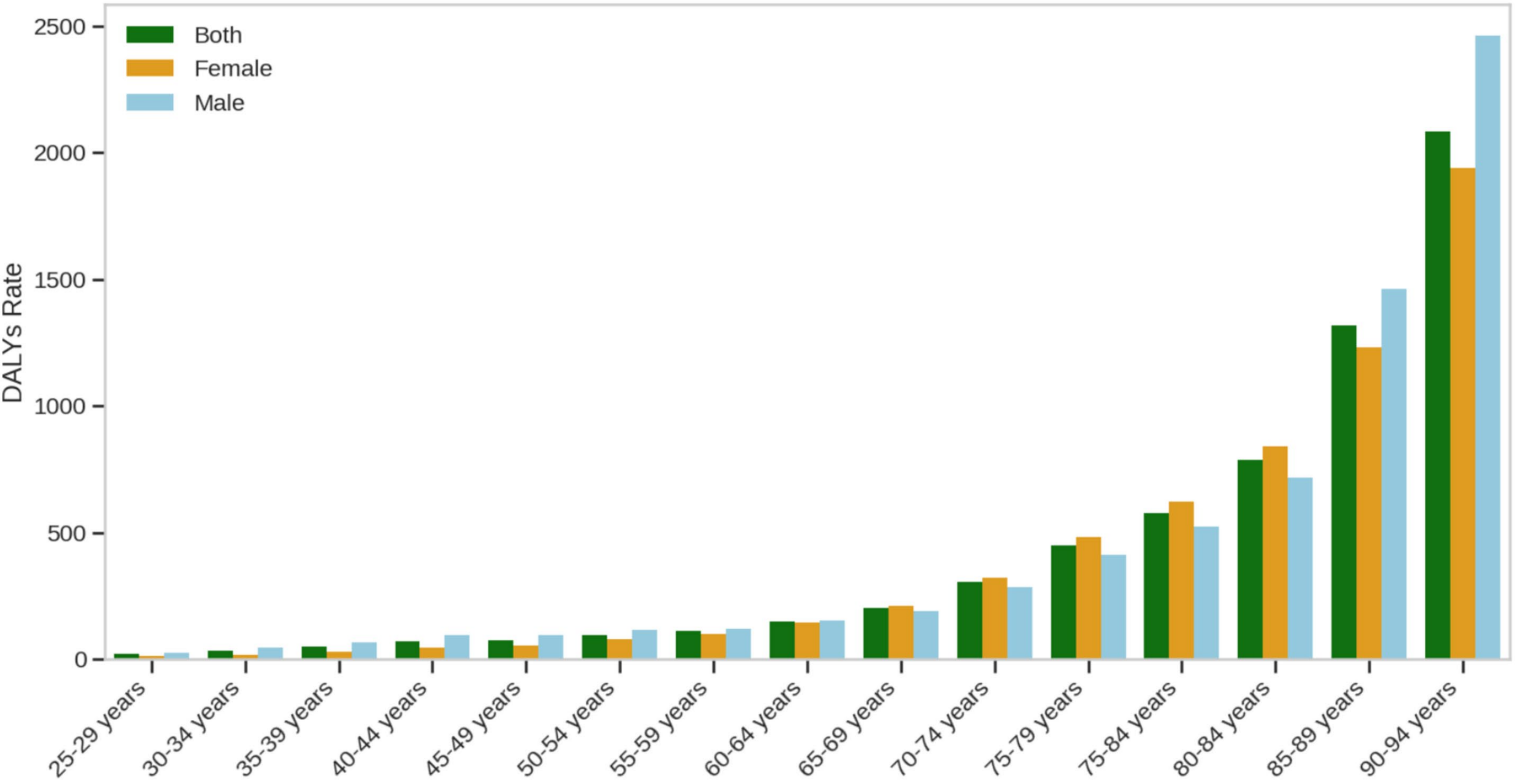
DALY rates due to ischemic heart disease attributable to insufficient omega-3 fatty acid intake in 2021 by gender and age group.

This study provides an in-depth analysis of the age and gender distribution characteristics of Disability-Adjusted Life Years (DALYs) due to ischemic heart disease (IHD) attributable to insufficient omega-3 fatty acid intake in China, revealing the multidimensional nature of the disease burden. The results (Figure 2) showed a significant age gradient effect in DALY rates, with rates steadily increasing from 15 to 16 per 100,000 in the 25–29 age group, reaching a key inflection point at 45–50 years. The DALY rate for middle-aged men approached 30 per 100,000, while for the elderly (70 years and older), it peaked at 475 per 100,000, illustrating the cumulative and dynamic nature of disease burden.

Gender analysis revealed a more complex health risk pattern. The average DALY rate for males (297 per 100,000) was significantly higher than that for females (269 per 100,000), with a broader range of data fluctuations, reflecting the high heterogeneity of health risks in men across different age groups. Key findings included: an accelerated increase in DALY rates during middle age (45–50 years), suggesting that this period represents a crucial window for health interventions; and a significant rise in DALY rates in the elderly (over 70 years), highlighting the risks of disease and functional loss faced by the elderly population.

It is noteworthy that the study data begins from the 25–29 age group, primarily reflecting the health burden in adults. This finding emphasizes the long-term importance of omega-3 fatty acid intake for adult health and provides scientific evidence for the development of targeted nutritional intervention strategies.

### 3.2 Trends in the disease burden of ischemic heart disease attributable to insufficient omega-3 fatty acid intake in Chinese adults from 1990 to 2021

**Figure 3:**
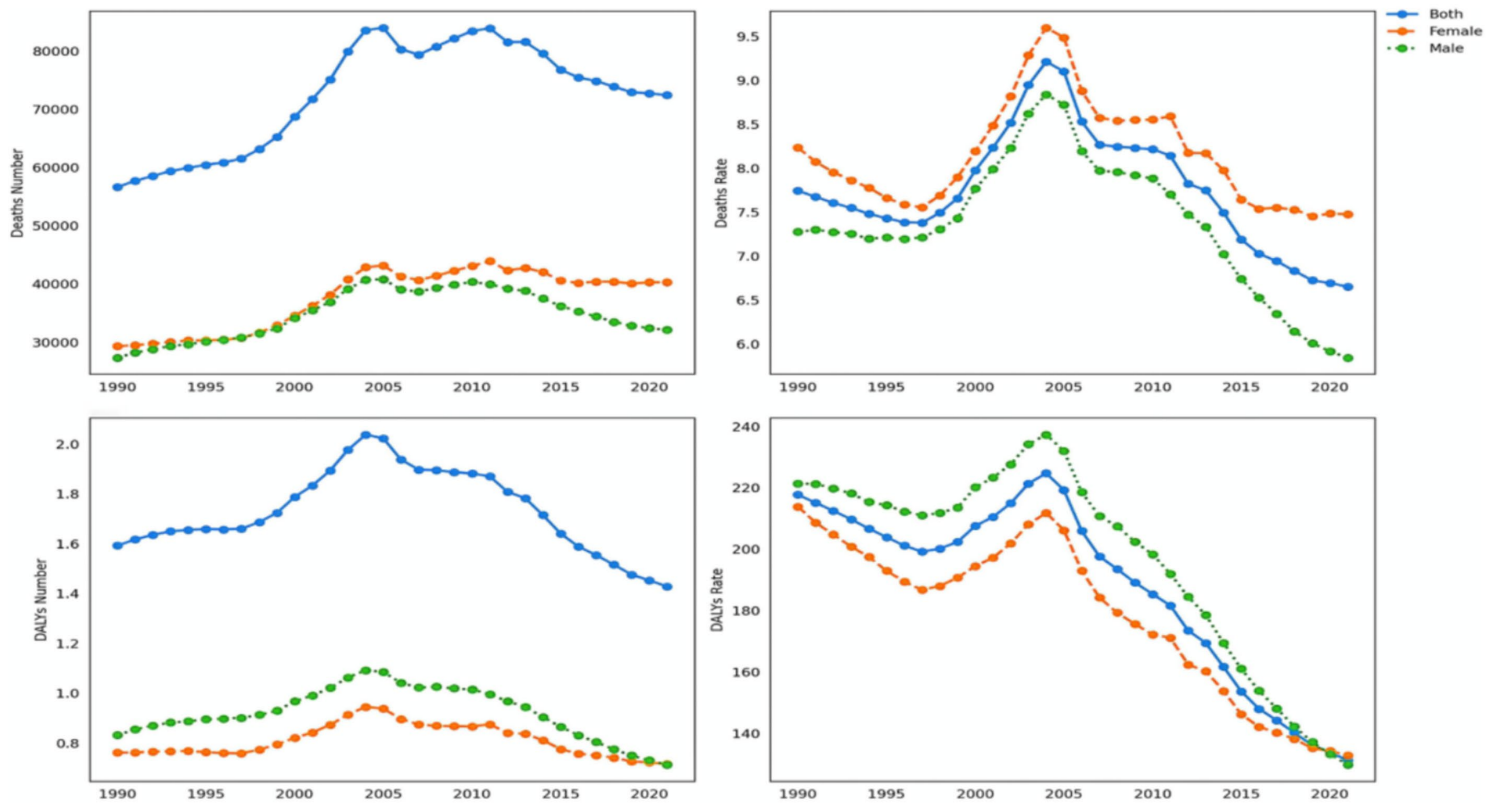
Trends in mortality and DALY rates due to ischemic heart disease attributable to insufficient omega-3 fatty acid intake in Chinese adults.

Over the past three decades, the disease burden of ischemic heart disease (IHD) attributable to insufficient omega-3 fatty acid intake in China has shown a complex evolutionary pattern (Figure 3). Regarding deaths, the total number increased from approximately 27,324 in 1990 to 32,098 in 2021, representing a 17.47% increase. Among these, male deaths rose from about 17,500 to 20,500 (an increase of 17.14%), while female deaths increased from 9,824 to 11,598 (an increase of 18.05%).

Changes in death rates revealed a significant downward trend. The overall death rate decreased from 7.28 per 100,000 in 1990 to 5.84 per 100,000 in 2021, a decline of 19.72%. For males, the death rate dropped from 9.52 per 100,000 to 7.41 per 100,000 (a decrease of 22.16%), while for females, the death rate decreased from 5.04 per 100,000 to 4.27 per 100,000 (a decline of 15.28%). The total number of Disability-Adjusted Life Years (DALYs) also exhibited a downward trend, decreasing from 831,327 in 1990 to 712,928 in 2021, a reduction of 14.24%.

The change in DALY rates was particularly notable, dropping from 221.39 per 100,000 in 1990 to 129.75 per 100,000 in 2021, a decrease of 41.40%. The male DALY rate fell from 285.67 per 100,000 to 167.41 per 100,000 (a decrease of 41.41%), while the female DALY rate decreased from 157.12 per 100,000 to 92.09 per 100,000 (a reduction of 41.37%). Throughout the study period, both male deaths and DALY counts consistently remained significantly higher than those for females, accounting for approximately 65–66% of the total. The male death rate and DALY rate were about 1.8–1.9 times those of females.

The overall trend showed a U-shaped pattern, manifested as follows: the number of deaths increased by 17.24%, the death rate decreased by 19.57%, DALY counts decreased by 14.36%, and DALY rates decreased significantly by 41.23%. This complex pattern of change likely reflects a combination of factors, including improvements in healthcare, lifestyle changes, and adjustments to nutritional intervention strategies. The study results not only reveal the long-term impact of insufficient omega-3 fatty acid intake on the disease burden of ischemic heart disease but also provide important scientific evidence for the development of targeted public health intervention strategies.

### 3.3 APC model analysis of the disease burden of ischemic heart disease attributable to insufficient omega-3 fatty acid intake in China from 1990 to 2021

#### 3.3.1 Net and local drift results of disease burden attributable to insufficient omega-3 fatty acid intake in China from 1990 to 2021

**Figure 4:**
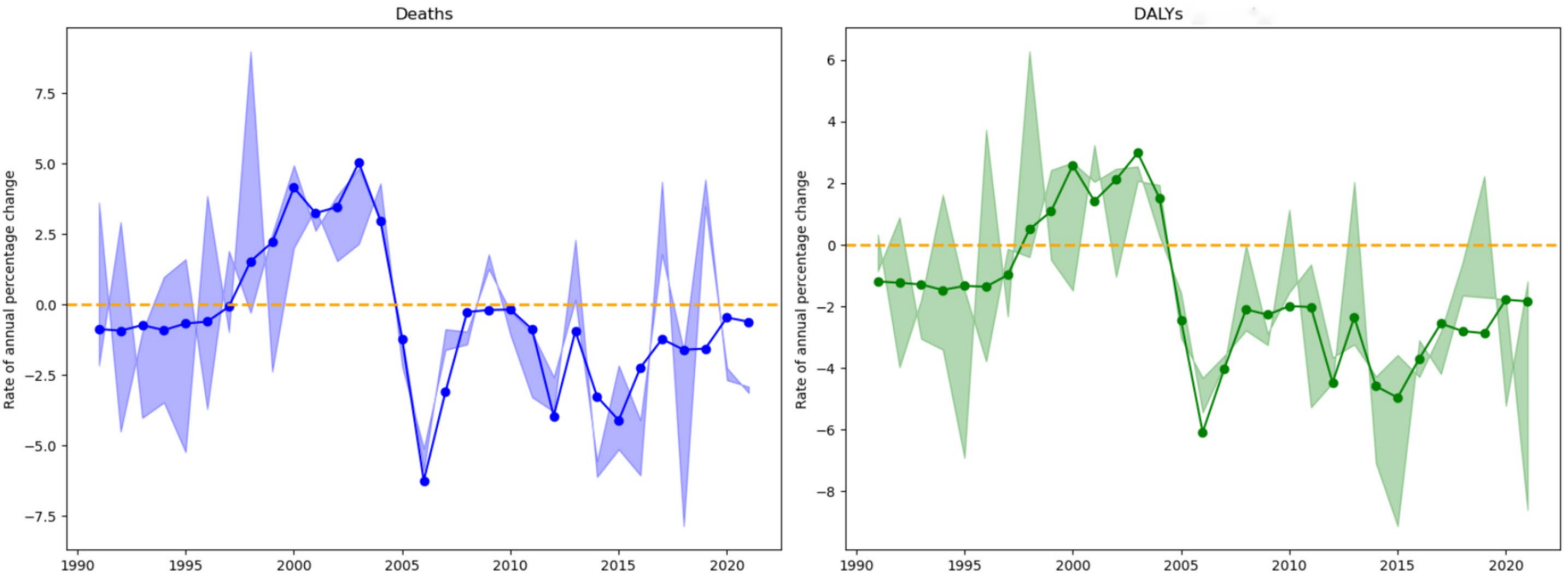
Net drift values of disease burden due to ischemic heart disease attributable to insufficient omega-3 fatty acid intake in China from 1990 to 2021.

Net drift is a key indicator used to assess the long-term evolutionary trend of a disease, reflecting the annual percentage change in deaths or disability-adjusted life years (DALYs) of a specific disease over the study period. Based on the analysis of data from 1990 to 2021 regarding ischemic heart disease attributable to insufficient omega-3 fatty acid intake in China (Figure 4), the study found that the net drift value for deaths was −0.32% (95% confidence interval: −0.63% to −0.01%). This indicates that the death rate decreased by an average of approximately 0.32% annually, and this change was statistically significant.

The net drift value for DALYs was −1.53% (95% confidence interval: −1.85% to −1.22%), suggesting that the DALY rate decreased by an average of approximately 1.53% per year. Compared to the death rate indicator, the decline in DALYs was more pronounced, and the statistical significance was clearer. These results highlight the substantial progress made in China in mitigating the burden of ischemic heart disease related to insufficient omega-3 fatty acid intake, particularly in terms of reducing DALYs. The negative net drift trend clearly indicates a continuous decline in the disease burden, providing important scientific evidence for future health intervention strategies.

**Figure 5:**
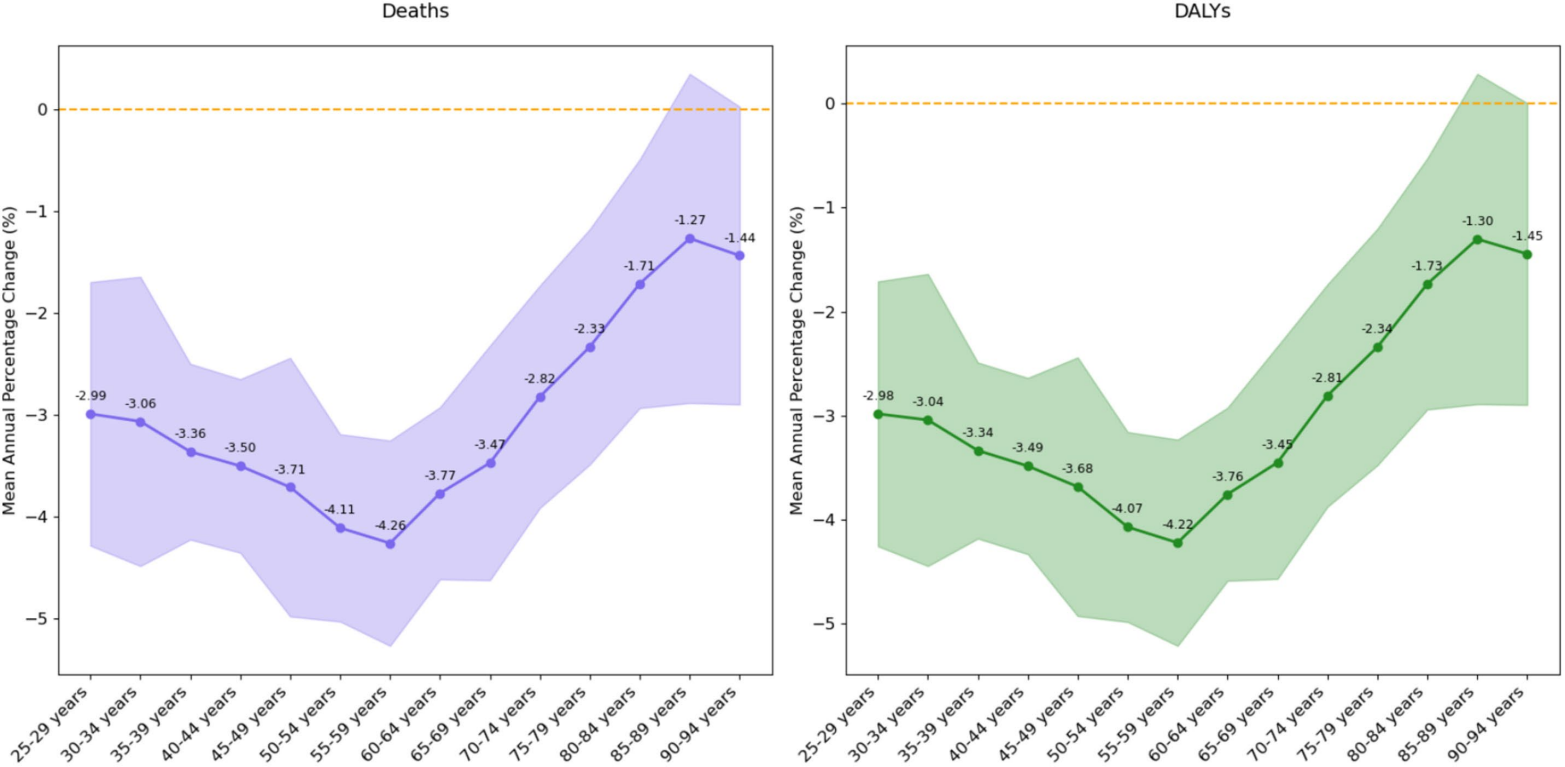
Local drift values of disease burden due to ischemic heart disease attributable to insufficient omega-3 fatty acid intake in China from 1990 to 2021.

Local drift values are statistical indicators used to describe the changes in disease deaths and disability-adjusted life years (DALYs) by calculating the annual percentage change in disease burden across different age groups. These values reveal the dynamic evolution characteristics of the disease at various stages of population development. The study provides a detailed age group classification to present the age-specific differences in disease burden (Figure 5).

For the young adult group (25–39 years), the average annual change rates were −3.14% for deaths and −3.12% for DALYs, with moderate variability (standard deviation approximately 3.3). Within this group, the 35-39-year-old age subgroup exhibited the most stable change (standard deviation 2.41), while the 30–34-year-old subgroup showed the most significant fluctuations (standard deviation 3.97). Overall, the young adult group displayed a relatively stable downward trend, with the lower bound of the 95% confidence interval significantly below −4.5%, indicating continuous improvement in disease burden for this age group.

The middle-aged group (40–59 years) showed the most stable and significant downward trend, with average annual change rates of −3.90% for deaths and −3.87% for DALYs, and the lowest variability (standard deviation approximately 2.8). All subgroups within this age range exhibited a sustained decline, with the 55–59-year-old subgroup showing the most significant decrease (−4.26% in deaths, −4.22% in DALYs). The confidence interval for this group was narrow, highlighting the high reliability of the estimates, especially during the period of 2005–2006, when the decline rate approached −10%. This finding not only underscores the significant progress made in reducing the disease burden in the middle-aged population but also provides important evidence for targeted public health interventions.

#### 3.3.2 Age-specific trends in death and DALY rates due to ischemic heart disease attributable to insufficient omega-3 fatty acid intake in China from 1990 to 2021

**Figure 6:**
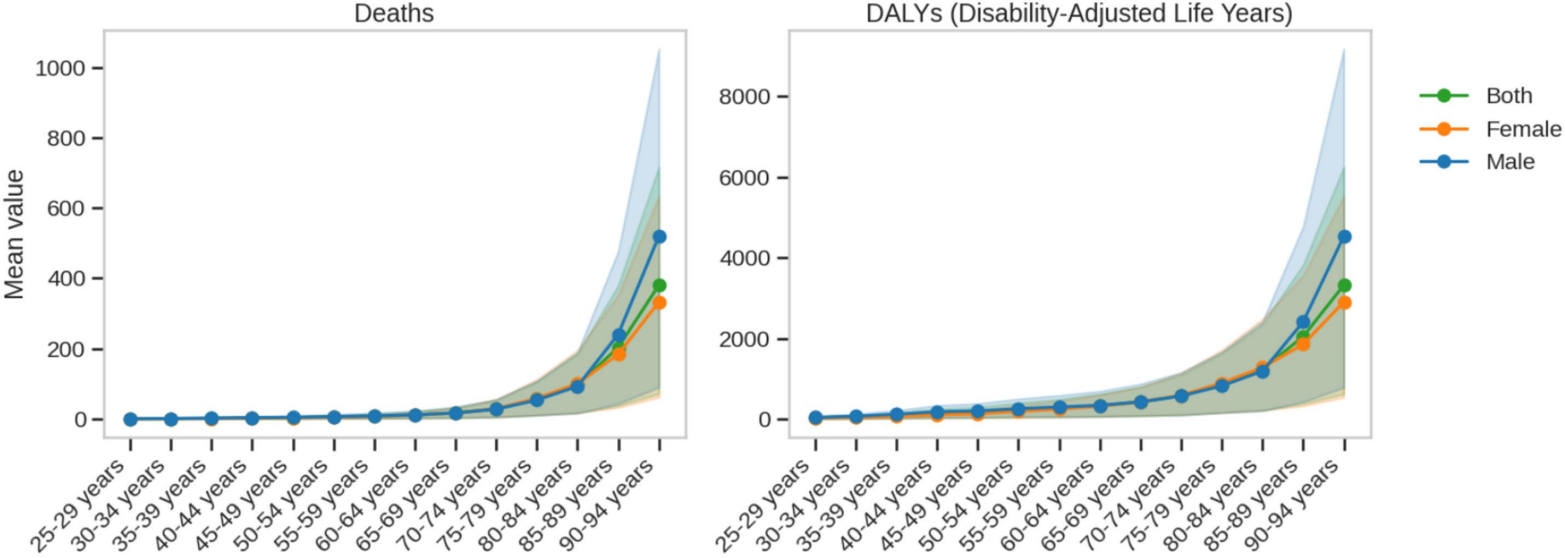
Age-specific trends in death and DALY rates due to ischemic heart disease attributable to insufficient omega-3 fatty acid intake from 1990 to 2021.

The study data revealed a significant age-progressive trend in death rates due to ischemic heart disease (Figure 6). In the 25–29 age group, the rate was only 0.56 per 100,000, after which it steadily increased. By the time individuals reached middle age (60–64 years), the rate rose to approximately 11.16 per 100,000. The rate further increased to 56.47 per 100,000 in the 75–79 age group, 97.36 per 100,000 in the 80–84 age group, and sharply rose to 380.69 per 100,000 in the 90–94 age group. This age-gradient effect was particularly pronounced in the elderly, with the rate of increase accelerating in the older age groups, reflecting the cumulative impact of aging on disease risk.

A significant gender difference was observed in ischemic heart disease death rates. The overall death rate for males (average: 70.80 per 100,000) was significantly higher than that for females (average: 53.73 per 100,000), with the gap becoming even more pronounced in middle to old age. The maximum value for males reached 519.78 per 100,000, while for females it was 332.38 per 100,000. This ongoing gender disparity suggests that men may face higher disease risks and health burdens at different age stages, which could be closely related to physiological mechanisms, lifestyle, and social factors.

DALY rates also demonstrated a significant age-gradient effect. In the 25–29 age group, the rate was only 35.45 per 100,000, after which it rapidly increased, reaching nearly the upper limit of 3,321.78 per 100,000 in the 90–94 age group, marking an approximately 94-fold increase. The overall average DALY rate was 701.76 per 100,000, with males averaging 820.38 per 100,000 (range: 42.76–4,532.02 per 100,000), and females averaging 649.70 per 100,000 (range: 27.83–2,901.43 per 100,000).

The trends in death and DALY indicators varied with age: death rates consistently increased linearly, while DALYs began to rise significantly in middle age (40–54 years) and continued to increase in old age, reflecting the multidimensional nature of the health burden. Gender analysis further revealed that males consistently had higher values for both indicators, particularly in middle and old age (45–74 years), with male DALY values approximately 26.3% higher than those of females.

These findings not only highlight the complex effects of insufficient omega-3 fatty acid intake on ischemic heart disease but also provide crucial scientific evidence for the development of more precise and stratified public health intervention strategies. The study emphasizes the importance of age- and gender-specific interventions, laying the foundation for personalized health management. By thoroughly analyzing the disease burden characteristics across different age groups and genders, this research offers valuable insights for precision medicine and public health policy formulation.

#### 3.3.3 Periodic trends in death and DALY rates due to ischemic heart disease attributable to insufficient omega-3 fatty acid intake in China from 1990 to 2021

**Figure 7:**
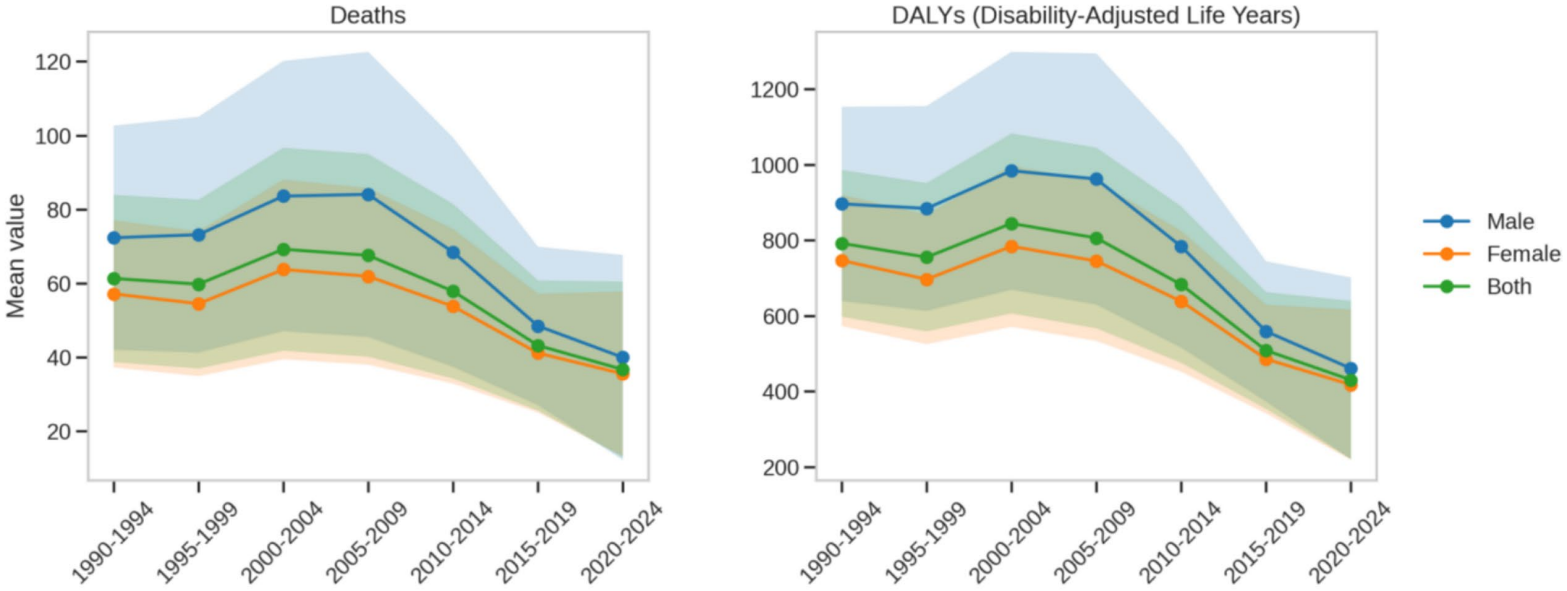
Periodic trends in death and DALY rates due to ischemic heart disease attributable to insufficient omega-3 fatty acid intake from 1990 to 2021.

In the context of omega-3 fatty acid deficiency, male ischemic heart disease death rates showed significant historical phase changes (Figure 7). In the early 1950s, the average death rate was as high as 150.0 per 100,000. With the continuous improvement of public health and medical technologies, by the period 1990–1994, the death rate decreased to 100.0 per 100,000, a reduction of approximately 33%. Corresponding to the death rate changes, the male disease burden (DALYs) also significantly decreased, from 350.0 per 100,000 in the early period to 250.0 per 100,000 in the recent period.

Female ischemic heart disease death rates also showed a declining trend, but the extent of change and the absolute values were significantly lower than those for males. In the early period, the average death rate was around 120.0 per 100,000, decreasing to 90.0 per 100,000 in the recent period, a reduction of about 25%. Correspondingly, the female disease burden (DALYs) decreased from 280.0 per 100,000 in the early period to 200.0 per 100,000 in the recent period. Compared to males, the changes in women’s health status over different historical periods were more stable, with less fluctuation in both death rates and disease burden.

A comparative analysis revealed that both males and females benefited from advances in public health and medical technologies, but the extent of this impact was significantly different. In the early period, male death (150.0 per 100,000) was significantly higher than that of females (120.0 per 100,000); in the recent period, male death (100.0 per 100,000) remained higher than female death (90.0 per 100,000). The patterns of disease burden (DALYs) followed a similar trend: early males (350.0 per 100,000) vs. females (280.0 per 100,000), recent males (250.0 per 100,000) vs. females (200.0 per 100,000).

This persistent gender difference suggests that males face higher cardiovascular health risks and are more sensitive to environmental changes across historical phases. The findings underscore the importance of developing gender-specific public health intervention strategies, particularly for male populations, focusing on omega-3 fatty acid nutritional interventions and cardiovascular health management. By revealing the changing characteristics of disease burden in males and females across different periods, this study provides important scientific evidence for precision health interventions and emphasizes the critical role of gender and historical context in disease burden assessments.

#### 3.3.4 Trends in death and DALY rates due to ischemic heart disease attributable to insufficient omega-3 fatty acid intake by birth cohort from 1990 to 2021 in China

**Figure 8:**
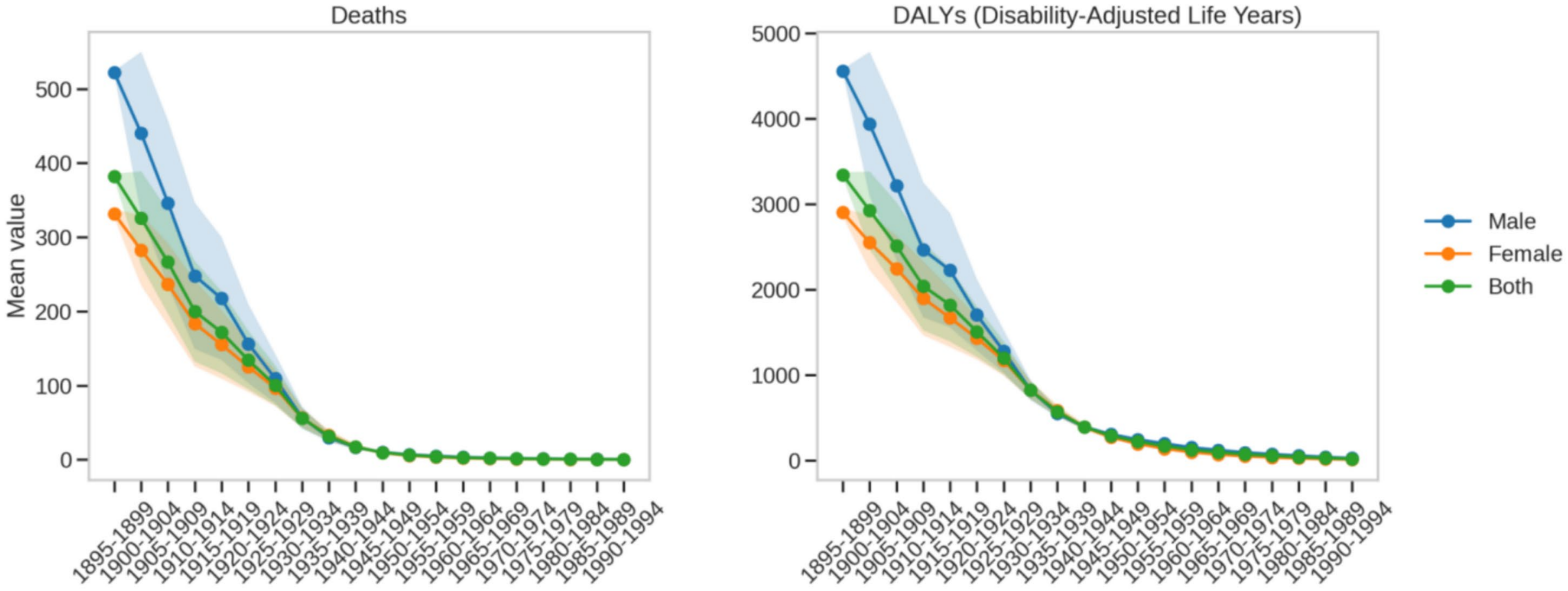
Trends in birth cohort changes in death and DALY rates due to ischemic heart disease attributable to insufficient omega-3 fatty acid intake from 1990 to 2021.

In the context of omega-3 fatty acid deficiency, intergenerational changes in male ischemic heart disease death rates show a highly significant improvement trend (Figure 8). For the early-born cohorts (1895–1899), the average death rate was as high as 3341.66 per 100,000. However, for the most recent cohort (1990–1994), this rate dramatically dropped to 20.98 per 100,000, with a linear regression slope of approximately −164.65. Corresponding to the death rate changes, the male disease burden (DALYs) sharply decreased from 3500.0 per 100,000 in the early-born cohort to 30.0 per 100,000 in the most recent cohort. This dramatic shift reflects revolutionary progress in public health, medical technology, and living conditions.

The intergenerational changes in female ischemic heart disease death rates also exhibit a significant improvement trend. For early-born female cohorts, the average death rate was about 3000.0 per 100,000, which decreased to between 15.0 and 25.0 per 100,000 in the most recent cohort (1990–1994). Similarly, the female disease burden (DALYs) decreased from 3200.0 per 100,000 in the early-born cohort to approximately 25.0 per 100,000 in the most recent cohort. This pattern mirrors that of males, demonstrating the significant health benefits that modern medical and living standards have brought to women.

A comparative analysis reveals that both males and females show a significant shift from high-risk to low-risk over generations, but with key differences. In terms of death rates, males decreased from 3341.66 per 100,000 to 20.98 per 100,000, while females dropped from approximately 3,000 per 100,000 to between 15 and 25 per 100,000. For disease burden (DALYs), males dropped from 3500.0 per 100,000 to 30.0 per 100,000, while females decreased from 3200.0 per 100,000 to around 25.0 per 100,000. Although both genders benefited from public health advancements, the absolute improvement for males is more pronounced numerically due to their higher initial risk.

This difference not only reflects the inherent physiological differences between genders but also highlights the importance of gender-specific public health intervention strategies. The findings underscore the need to consider gender-specific approaches in omega-3 fatty acid nutrition and cardiovascular health management, and to develop more precise interventions. By analyzing the changes in disease burden across different birth cohorts, this study provides important scientific evidence for understanding long-term health trends and informing targeted public health policies.

## 4 Discussion

This study systematically assesses the spatiotemporal distribution characteristics and long-term evolution patterns of ischemic heart disease (IHD) burden attributable to insufficient omega-3 fatty acid intake among Chinese adults. Through multidimensional spatiotemporal analysis, the research not only quantifies the relationship between nutritional deficiencies and cardiovascular health but also reveals the complex bio-social interaction mechanisms involved in the dynamic evolution of disease burden, providing important scientific evidence for precision nutrition interventions.

### 4.1 Biological basis of age- and sex-specific risks

The study observed that death increases exponentially with age (a 684-fold increase from 25–29 years to 90–94 years), which is highly consistent with the cumulative pathological effects of atherosclerosis (21). The age-dependent acceleration of low-density lipoprotein (LDL) oxidation modification and the inflammatory cascade (22) may amplify the negative effects of omega-3 fatty acid deficiency on the metabolism balance of eicosanoids (23, 24). This finding reveals the cumulative effect of aging on cardiovascular disease risk (25), providing important insights into the relationship between nutritional deficiencies and disease progression.

The gender difference analysis revealed more complex biological mechanisms. Death rates in middle-aged men (30–50 years) were significantly higher than those in women (54–78% higher), reflecting the key role of gender in cardiovascular health (26). Androgens regulate hepatic lipoprotein lipase activity, significantly increasing apolipoprotein B secretion and accelerating lipid metabolism abnormalities (27). At the same time, high-intensity occupational stress, which leads to sustained sympathetic nervous system activation, further accelerates endothelial dysfunction and oxidative stress processes (28). The significantly higher smoking rates among Chinese men compared to women (29), coupled with the negative interaction effects of omega-3’s anti-inflammatory action (30), further amplify cardiovascular disease risk.

The death rate for women aged 70–84 surpasses that of men, marking a turning point that raises more scientific questions worth further investigation. The sharp decline in estrogen levels after menopause leads to dysfunction of high-density lipoprotein cholesterol (HDL-C), weakening its original vascular protective effects (31). Meanwhile, the high prevalence of sarcopenia exacerbates the potential damage caused by Omega-3 fatty acid deficiency to myocardial energy metabolism (32). This phenomenon not only contrasts with the cardiovascular risk pattern observed in elderly women in Japan (33), but also suggests the existence of a unique nutrition-aging interaction pathway in the Chinese population. According to age effect estimates, the mortality rate in the 90–94 age group peaked at 3,806.93 per 100,000 population. Given that mortality among individuals aged 80 years and older is influenced by numerous confounding factors, these findings should be interpreted with particular caution in the oldest-old population.

Omega-3 fatty acids exert cardioprotective effects through multiple molecular mechanisms, including modulation of inflammatory responses, improvement of endothelial function, and regulation of lipid metabolism (34, 35). In terms of inflammation, omega-3 fatty acid derivatives—such as resolvins and protectins—suppress the NF-κB signaling pathway and reduce the expression of pro-inflammatory cytokines, including IL-6 and TNF-*α*. Regarding lipid metabolism, omega-3 fatty acids activate the transcription factors PPARα/*γ*, thereby enhancing fatty acid *β*-oxidation, inhibiting lipogenesis, promoting high-density lipoprotein (HDL) formation, and reducing apolipoprotein B levels (36). With respect to endothelial function, EPA and DHA upregulate endothelial nitric oxide synthase (eNOS) activity, increase nitric oxide (NO) bioavailability, and suppress endothelin-1 levels, thereby maintaining vascular homeostasis (36). Additionally, omega-3 fatty acids confer protective effects by inhibiting platelet aggregation, stabilizing atherosclerotic plaques, and improving myocardial membrane fluidity and ion channel function (37). With advancing age, the regulatory efficiency of these molecular pathways generally declines, while hormonal changes—such as androgen-induced lipid dysregulation and diminished estrogenic protection—further disrupt the balance of these mechanisms (38). Integrating these molecular pathways provides mechanistic context for the observed age- and sex-specific patterns of disease burden.

The findings highlight the importance of developing age- and sex-specific personalized nutrition intervention strategies. The traditional “one-size-fits-all” approach to health intervention is no longer sufficient to address such complex physiological changes. Future nutrition interventions should be more precise, taking into account the specific physiological needs of different age groups and genders, and building more personalized cardiovascular health management strategies.

### 4.2 Epidemiological drivers of disease burden evolution

Between 1990 and 2021, the disease burden of ischemic heart disease (IHD) in China exhibited complex demographic transition characteristics. Despite a 19.72% decrease in age-standardized death, the absolute number of deaths increased by 17.47%. This phenomenon vividly illustrates the delicate balance between population aging and risk factor control. This trend is highly consistent with the heart disease evolution pattern in East Asia as observed in the Global Burden of Disease (GBD) study; however, the decrease in China’s DALY rate (−41.40%) is significantly higher than the global average (−28.6%), potentially reflecting the positive dietary transformation over the past three decades (39), particularly the steady increase in the consumption of Omega-3-rich foods, such as deep-sea fish.

The Age-Period-Cohort (APC) model further revealed the multidimensional driving mechanisms behind the evolution of disease burden. At the age effect level, the death rate for the 90-94-year-old age group reached 3806.93 per 100,000. This data not only reflects the challenges posed by extreme population aging but also suggests that the bioavailability of Omega-3 fatty acids in older populations significantly declines, which may exacerbate the processes of ventricular remodeling and cardiovascular function degradation (40).

Period effect analysis identified a critical turning point: in 2005–2006, there was a significant −10% decrease in DALY rates for the middle-aged group. This timing coincided with the update of the “Cardiovascular Disease Secondary Prevention Guidelines” and the inclusion of EPA/DHA supplements in the national health insurance policy. This suggests that targeted public health policies can yield significant health intervention effects in a relatively short period of time.

Cohort effect analysis further revealed the long-term impact of lifelong nutrition on cardiovascular health. From the 1895–1994 birth cohorts, death rates dropped by as much as 99.3%, and this substantial change is likely closely linked to systematic improvements in early-life nutritional environments. For example, the 1995 iodized salt policy not only improved trace element intake but may also have had long-term protective effects on vascular elasticity and cardiovascular system development.

These findings collectively point to an important conclusion: the evolution of disease burden is a complex result of the interplay between biological, socioeconomic, and public health policy factors. The traditional single-intervention approach is no longer sufficient to address such complex health challenges, highlighting the need to establish more dynamic and precise public health intervention paradigms.

### 4.3 Strengths and limitations of the study

This study pioneered a multidimensional spatiotemporal analytical framework by integrating the Age–Period–Cohort (APC) model with burden of disease assessment to systematically delineate the evolving trajectory of ischemic heart disease (IHD) burden attributable to omega-3 fatty acid deficiency in China from 1990 to 2021. The analysis precisely disentangled the independent contributions of age, period, and cohort effects, revealing an intergenerational transmission pattern of nutrition-related cardiovascular risk, and thereby laying a theoretical foundation for life-course health intervention strategies. Methodological innovation was reflected in the use of quantitative indices such as net drift and local drift, enabling precise measurement of spatiotemporal heterogeneity in disease burden. The study found that the disability-adjusted life year (DALY) rate in middle-aged adults (40–59 years) declined by an average of 3.87% per year, providing a dynamic metric for evaluating the effectiveness of public health policies. Furthermore, the analysis identified critical intervention windows (e.g., a DALY rate inflection point at ages 45–50) and high-risk subpopulations (including middle-aged men and older women), and constructed a stratified prevention framework based on the omega-3 index, effectively supporting the implementation pathway for the cardiovascular goals of the “Healthy China 2030” initiative.

Despite these significant contributions, the study has several limitations in exposure assessment and data application. First, omega-3 fatty acid intake was estimated using indirect methods rather than direct measurement of serum EPA/DHA levels, which may not capture individual differences in bioavailability and could introduce exposure misclassification. Second, the analysis did not fully account for the impact of traditional Chinese cooking practices (e.g., high-temperature frying) on fatty acid oxidation losses, potentially affecting intake estimates. Although the study applied the standardized confounder adjustment models from the GBD framework, it was unable to incorporate some potentially important confounders, such as APOE genetic polymorphisms and gut microbiota composition, which may influence risk attribution accuracy. Due to the ecological study design, the analysis could not establish a dose–response relationship at the individual level, and the association between omega-3 deficiency and mortality relied primarily on theoretically derived relative risk (RR) estimates. Moreover, the data were limited to pre-2021, and thus do not reflect changes in dietary patterns following the COVID-19 pandemic. The study also did not systematically assess the potential risk of heavy metal exposure (e.g., methylmercury) associated with marine-derived omega-3 fatty acids, which may overestimate the net health benefits of deep-sea fish consumption.

Although animal intervention studies [e.g., (41)] have clearly demonstrated that omega-3 polyunsaturated fatty acid supplementation can significantly improve various cardiovascular risk markers—such as lowering the ApoB100/ApoA1 ratio, reducing glycated hemoglobin, and enhancing antioxidant capacity—large-scale meta-analyses [e.g., (11)] have suggested that omega-3 supplementation does not significantly reduce major cardiovascular events in randomized controlled trials (RCTs). This inconsistency underscores that even though RCTs offer the highest level of causal evidence, their results can still be influenced by population heterogeneity, intervention dosage, and adherence. Therefore, the findings of this study should also be interpreted with caution.

Future research should integrate data from large prospective cohorts such as the China Cardiovascular Health Study and the China Health and Retirement Longitudinal Study (CHARLS), and adopt more refined exposure assessment tools (e.g., population-specific food frequency questionnaires and serum fatty acid profiling), while accounting for the impact of regional cooking practices on fatty acid oxidation. Studies should differentiate the risk contributions of plant-derived alpha-linolenic acid (ALA) versus marine-derived EPA/DHA to ischemic heart disease, and conduct comprehensive sensitivity analyses to test the robustness of findings after adjusting for lifestyle factors such as physical activity, alcohol consumption, and ultra-processed food intake. In addition, exploring specific gene–environment interactions will help unravel the complex relationship between omega-3 fatty acids and IHD risk. Future models should also incorporate medication use (e.g., statins) and comorbidities (e.g., diabetes, hypertension) to enhance the precision of risk attribution and support the development of population-specific precision nutrition strategies for cardiovascular disease prevention in China.

## 5 Conclusion

This study is the first to construct a lifetime risk map of Omega-3 deficiency and IHD burden in the Chinese population, revealing key age-gender interaction nodes and providing evidence-based support for precision nutrition interventions. In the dual challenges of population aging and the increasing burden of metabolic diseases, optimizing Omega-3 intake strategies will become an important complement to the cardiovascular disease prevention and control system.

## Data Availability

The datasets presented in this study can be found in online repositories. The names of the repository/repositories and accession number(s) can be found at: https://vizhub.healthdata.org/gbd-results/.

## Footnotes

^1^ https://ghdx.healthdata.org/gbd-results-tool
